# RETRACTION: Effect of Probiotics and Synbiotics on Complications of Wound Infection after Colorectal Surgery: A Meta‐Analysis

**DOI:** 10.1111/iwj.70407

**Published:** 2025-03-21

## Abstract

**RETRACTION:** JiangJ. and RenF. , “Effect of Probiotics and Synbiotics on Complications of Wound Infection after Colorectal Surgery: A Meta‐Analysis,” International Wound Journal 21, no. 4 (2024): e14838, 10.1111/iwj.14838.38577937 PMC10996049

The above article, published online on 05 April 2024, in Wiley Online Library (http://onlinelibrary.wiley.com/), has been retracted by agreement between the journal Editor in Chief, Professor Keith Harding; and John Wiley & Sons Ltd. Following an investigation by the publisher, all parties have concluded that this article was accepted solely on the basis of a compromised peer review process. The editors have therefore decided to retract the article. The authors disagree with the retraction.

**RETRACTION:** J. Jiang and F. Ren , “Effect of Probiotics and Synbiotics on Complications of Wound Infection after Colorectal Surgery: A Meta‐Analysis,” International Wound Journal 21, no. 4 (2024): e14838, 10.1111/iwj.14838.38577937 PMC10996049

The above article, published online on 05 April 2024, in Wiley Online Library (http://onlinelibrary.wiley.com/), has been retracted by agreement between the journal Editor in Chief, Professor Keith Harding; and John Wiley & Sons Ltd. Following an investigation by the publisher, all parties have concluded that this article was accepted solely on the basis of a compromised peer review process. The editors have therefore decided to retract the article. The authors disagree with the retraction.

